# Behavioral responses of wolves to roads: scale-dependent ambivalence

**DOI:** 10.1093/beheco/aru134

**Published:** 2014-08-20

**Authors:** Barbara Zimmermann, Lindsey Nelson, Petter Wabakken, Håkan Sand, Olof Liberg

**Affiliations:** ^a^Faculty of Applied Ecology and Agricultural Sciences, Hedmark University College, Evenstad, N-2480 Koppang, Norway and; ^b^Department of Ecology, Grimsö Wildlife Research Station, Swedish University of Agricultural Science, SE-73091 Riddarhyttan, Sweden

**Keywords:** *Canis lupus*, functional response, movement, resource selection, road, step selection function, travel speed.

## Abstract

Throughout their recent recovery in several industrialized countries, large carnivores have had to cope with a changed landscape dominated by human infrastructure. Population growth depends on the ability of individuals to adapt to these changes by making use of new habitat features and at the same time to avoid increased risks of mortality associated with human infrastructure. We analyzed the summer movements of 19 GPS-collared resident wolves (*Canis lupus* L.) from 14 territories in Scandinavia in relation to roads. We used resource and step selection functions, including >12000 field-checked GPS-positions and 315 kill sites. Wolves displayed ambivalent responses to roads depending on the spatial scale, road type, time of day, behavioral state, and reproductive status. At the site scale (approximately 0.1 km^2^), they selected for roads when traveling, nearly doubling their travel speed. Breeding wolves moved the fastest. At the patch scale (10 km^2^), house density rather than road density was a significant negative predictor of wolf patch selection. At the home range scale (approximately 1000 km^2^), breeding wolves increased gravel road use with increasing road availability, although at a lower rate than expected. Wolves have adapted to use roads for ease of travel, but at the same time developed a cryptic behavior to avoid human encounters. This behavioral plasticity may have been important in allowing the successful recovery of wolf populations in industrialized countries. However, we emphasize the role of roads as a potential cause of increased human-caused mortality.

## Lay Summary

We studied how wolves in Scandinavia respond to roads built to ease human travel but degrading habitat quality for many wildlife species. Wolves responded with ambivalence: They both selected and avoided roads, all depending on the spatial and temporal scale and their behavioral status. To understand the multi-scale effects of human infrastructure on animal behavior is important with regard to the recent come-back of many wildlife species to now industrialized countries.

## INTRODUCTION

Roads are man-made habitat features that are hardly comparable to any natural habitat: they are linear, have an open canopy, a hard surface, and often have parallel open-canopy strips with ground-cover vegetation on both sides. Connected with other roads, they form a network causing fragmentation of natural habitats. Roads are among the most recent of man-made habitat alterations, having spread dramatically during the past century following the development of motor vehicles (Huston 2005). Reviews of ecological effects of roads on wildlife populations highlight the direct mortality caused by collisions with vehicles and the indirect alteration of individual behavior due to habitat loss, fragmentation, and increased human access (Forman and Alexander 1998; Coffin 2007; Fahrig and Rytwinski 2009; Benitez-Lopez et al. 2010). Increased access implies higher human-caused disturbance and predation risk as perceived by wildlife, thereby linking the two main types of human impact, habitat alteration and hunting. Our study aims to explore this link and its consequences for the behavioral ecology of the wolf, a top predator which is currently re-covering in many European countries (Linnell et al. 2005).

The wolf is a pack-living, highly mobile species that defends large pack territories by frequent scent-marking (Mech and Boitani 2003). Roads may therefore be a positive new addition to the landscape of wolves. Indeed, roads have been shown to ease travel for wolves (Musiani et al. 1998; James 1999; Whittington et al. 2005; Eriksen et al. 2009; Gurarie et al. 2011; Muhly et al. 2011). Roads can facilitate territorial patrolling and serve as distinct features for scent marking (Zub et al. 2003; Barja et al. 2004). Roads can lead to increased encounter rates between wolves and their prey and therefore increase kill rates (James and Stuart-Smith 2000; Hebblewhite et al. 2005a; Whittington et al. 2011). As road sides are typical edge habitats with plant communities in early succession, they may provide minerals and energy-rich food for grazing and browsing prey species of wolves (Forman and Alexander 1998; Laurian et al. 2008; Rea et al. 2010) close to shelter habitat.

On the other hand, roads have been shown to increase mortality of wolves directly due to traffic accidents and indirectly by increasing access for hunters and poachers (Thiel 1985; Mech et al. 1988; Person and Russell 2008). Fragmentation decreases the availability of undisturbed habitat (Jędrzejewski et al. 2001) whereas large roads can affect wolf movement (Whittington et al. 2004) and in some cases act as barriers that limit dispersal and distribution on the population level (Alexander and Waters 2000), but see also Blanco et al. (2005). Several studies have shown that the occurrence of wolf territories is higher in areas with low densities of roads and built-up areas (Thiel 1985; Mladenoff et al. 1995; Kaartinen et al. 2005; Karlsson et al. 2007; Jędrzejewski et al. 2008). The use of roads by wolves is likely constrained by the extent of human activity which may be perceived as an indicator of the risk of human-caused mortality. Previous studies have concluded that wolves prefer to use roads and trails with low human use (Kunkel and Pletscher 2000; Whittington et al. 2005) or during times of low human activity, such as during night as opposed to day or winter as opposed to summer (Theuerkauf et al. 2003a; Theuerkauf 2009). Preference for forest roads decreases with increasing road density within wolf home ranges (functional response, Houle et al. 2010). Reproducing wolves and their pups are likely most vulnerable to humans and other large predators during the summer season because movements are centered round a den site and later rendezvous sites (Jędrzejewski et al. 2001; Schmidt et al. 2008; Tsunoda et al. 2009). Survival of both adults and pups may be severely affected if these sites are detected. As a result, such sites are often located far from sources of human disturbance (Theuerkauf et al. 2003b; Capitani et al. 2006; Person and Russell 2009).

In summary, roads pose a trade-off for wolves between human-induced negative effects (disturbance and increased mortality) and the positive effects resulting from increased ease of travel, efficient scent-marking, and access to prey. Our study examines ambivalent responses toward roads at different spatial and temporal scales from single wolf steps up to the landscape level for wolves in Scandinavia. After the functional extinction of the Scandinavian wolves in the 1960s (Wabakken et al. 2001), a couple of immigrant wolves from Finland or Russia founded today’s population on the Scandinavian Peninsula in the early 1980s (Vilà et al. 2003). The population increased rapidly after 1990 (Wabakken et al. 2001) and totalled 33 family groups and 27–28 scent-marking pairs of wolves in winter 2011/2012 (Wabakken et al. 2012). Although listed as critically endangered in Norway (Kålås et al. 2010) and endangered in Sweden (Gärdenfors 2010), the wolf is still subject to both legal and illegal hunting in both countries. Poaching is assumed to account for half of all wolf mortality (Liberg et al. 2012). Conflicts regarding depredation of livestock, perceived competition for game species, and the loss of hunting dogs to wolves, all lead to an acceptance of poaching, especially in rural areas with free-ranging livestock and strong hunting traditions (Gangaas et al. 2013). The dense network of gravel roads created during recent decades for forest exploitation is facilitating the access of poachers to remote areas. Traffic accidents also contribute substantially to wolf mortality in Scandinavia (Wabakken et al. 2001; Morner et al. 2005; Liberg et al. 2012) despite low densities of main roads and humans (Wabakken et al. 2001; Karlsson et al. 2007).

Habitat selection is a process acting at different spatial scales. According to Johnson’s (1980) classification, animals choose locations at the landscape level (population range, first order), at the home range level within the population range (second order), at the patch level within the home range (third order), and at the site level within a patch (fourth order). Our study focused on the home range, patch, and site levels. We first tested the ease-of-travel hypothesis by predicting that the travel speed of wolves is higher on roads as compared to off roads. Secondly at the site scale (approximately 0.1 km^2^), we examined how road type, time of day, and wolf behavioral state affect road use and the distance wolves stay from roads (Johnson’s fourth order). We predicted that wolves 1) prefer to use gravel roads and avoid main roads; 2) use roads more often and are closer to roads during night than daytime; and 3) use roads mainly while traveling and avoid being close to roads while handling prey and resting. Thirdly, we examined whether the wolves’ selection of patches within the home range (third order, spatial scale of 10 km^2^) was dependent on road density. We expected wolves to prefer areas of low road density in order to avoid human disturbance, and we expected breeding wolves to show a stronger avoidance than nonbreeders. Finally, at the home range scale (second order, approximately 1000 km^2^), we tested whether road use by wolves was a function of road density. We predicted a functional response due to human disturbance, that is wolves living in territories with high road densities use roads relatively less frequently than wolves in territories with low road densities. Our study focused on the summer period because 1) all gravel roads are available for wolves and people as opposed to winter when only some parts of gravel roads are snow-ploughed, and 2) breeding wolves may be more constrained during this period when movements are restricted to denning or rendezvous sites.

## MATERIALS AND METHODS

### Study area

**Figure 1:**
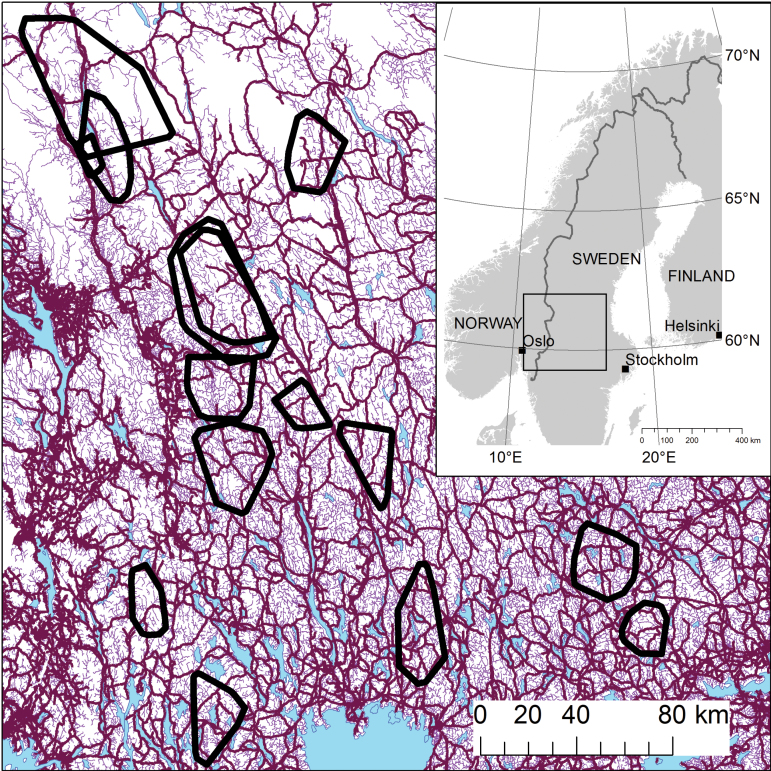
Location of wolf territories included in this study (black outlines), and all main roads (bold purple lines) and gravel roads (thin purple lines) within the study area. The study area comprised most of the wolf breeding range on the Scandinavian Peninsula (inset).

This study was carried out within the wolf breeding range in south-central parts of the Scandinavian Peninsula that is Sweden and Norway (Figure 1; 59–62°N, 10–15°E, approximately 100000 km^2^). The wolf territories were primarily covered by boreal coniferous forest (mean ± *SE*: 81.7±1.3% for *n* = 14 territories) dominated by Scots pine (*Pinus sylvestris* L.) and Norway spruce (*Picea abies* L.), with some deciduous species, of which birch (*Betula pubescens* Ehrh.) and aspen (*Populus tremula* L.) were most abundant. Mire was the second most frequent land cover type within the wolf territories (10.5±1.2%), followed by water (4.6±0.8%), agricultural fields (1.6±0.6%), open areas (e.g. mountains, boulder fields; 1.4±0.9%), and built-up areas (0.2%). The density of main roads within territories averaged 0.19±0.02 km/km^2^, and the maximum distance to main roads ranged from 3.72 to 14.88 km (Table 1). A large network of gravel roads has been created due to extensive commercial logging and forest management practices (Sand et al. 2008) (Figure 1). Gravel road densities in the territories were on average 4.6 times higher than main road densities and the maximum distance to gravel roads within territories ranged from 1.25 to 6.09 km (Table 1). Human density within the distribution of the Scandinavian wolf population is low, including vast areas with <1 person per km^2^ (Wabakken et al. 2001). House densities within the territories averaged 3.0±0.4 per km^2^.

**Table 1:** Wolf territories with area (100% MCP of all hourly GPS-positions during the study periods per territory and year), road densities and maximum distances to gravel roads and main roads

Territory	Year	Number of study periods	Number of data sets	Area km^2^	Road density km/km^2^		Max distance to road km	
					Main road	Gravel road	Main road	Gravel road
Bograngen	2003	2	2	1595	0.16	0.83	8.59	2.51
Djurskog	2004	2	2	313	0.14	0.73	6.11	1.40
Forshyttan	2005	1	1	676	0.35	1.05	4.49	1.63
Fulufjellet	2010	2	4	544	0.21	0.53	6.74	4.03
Glaskogan	2002	3	3	509	0.23	1.01	6.61	1.86
Gräsmark	2006	1	2	820	0.25	1.17	6.04	1.89
Gråfjellet	2003	2	4	633	0.07	0.89	10.65	2.71
	2004	1	1	102	0.02	0.89	5.71	2.07
Halgån	2003	2	2	453	0.21	0.8	4.25	1.82
Juvberget	2007	1	2	1038	0.16	0.74	8.59	2.51
Kloten	2009	1	2	616	0.26	1.16	5.2	1.25
Koppang	2004	2	4	2105^a^	0.14	0.53	14.89	6.09
Nyskoga	2003	1	1	261	0.20	0.82	5.10	1.71
Rotna	2004	1	1	631	0.17	1.18	6.89	1.52
Uttersberg	2005	2	2	304	0.29	0.93	3.72	1.39
Sum		24	33					
Mean				707	0.19	0.88	6.91	2.29
*SE*				136	0.02	0.05	0.74	0.33

^a^In period 1 (June 14–July 05) with pups: 402 km^2^; In period 2 (August 19–September 06) when pups lost: 1983 km^2^.

Moose (*Alces alces* L.) are the most important prey of wolves in Scandinavia, with a population density of approximately 1–2 moose/km^2^ in summer. For Scandinavian wolf packs, moose represent more than 95% of the food biomass in summer (Sand et al. 2008). Other ungulate prey are roe deer (*Capreolus capreolus* L.), semi-domestic and wild reindeer (*Rangifer tarandus* L.), red deer (*Cervus elaphus* L.), and domestic sheep (*Ovis aries* L.). Smaller prey are also available for the wolf, including beaver (*Castor fiber* L.), badger (*Meles meles* L.), mountain and European hares (*Lepus timidus* L., *Lepus europeus* Pallas), capercaillie (*Tetrao urogallus* L.), and black grouse (*Lyrurus tetrix* L.) (Sand et al. 2008).

### Study animals and period

As part of the Scandinavian Wolf Research Project (SKANDULV), the data for this study were collected on 19 adult scent-marking wolves resident in 14 territories between 2002 and 2010 (Table 1). The wolves were immobilized from a helicopter following standard procedures (Sand et al. 2005; Eriksen et al. 2011; Kreeger and Arnemo 2012) and equipped with a GPS neck collar (GPS-Simplex, Web-Direct, or Tellus by Followit, Sweden, or GPS-Plus by Vectronic Aerospace, Germany). The study included 24 study periods of 8–29 days between June 1 and September 29. In nine study periods, both the adult male and female were GPS-collared, resulting in 33 individual data sets (Table 1). GPS-collars were programmed for hourly (8 data sets) or half-hourly (25 data sets) positioning intervals. GPS wolf position data for this study originated in a study of summer kill rates (Sand et al. 2008), but we included additional, more recent time periods from two territories. During the study periods, eight of the territories had breeding wolves and in the other five the wolves were nonbreeding. In one territory, the adult wolves bred successfully but lost their pups after the first study period, and we therefore treated them as nonbreeders for the second study period (Koppang, Table 1).

### GPS positions and cluster definition

**Figure 2:**
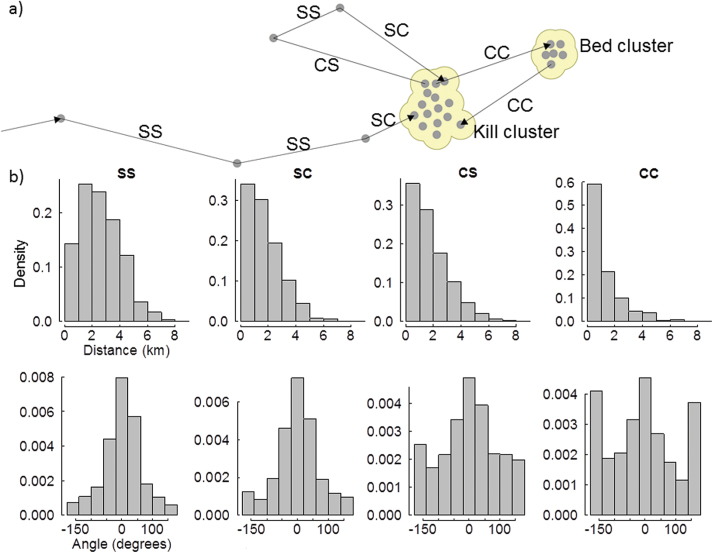
Movement analysis of GPS-positioning data of Scandinavian wolves showing how positions were separated into single positions >200 m from the nearest position, and cluster positions (a) and the step category-specific frequency distributions of step-lengths and turning angles used to create 10 random steps for each real step (b), where steps were categorized as: steps from single to single position (SS), single to cluster (SC), cluster to single (CS), and cluster to cluster (CC).

Our original data sets consisted of 13188 hourly and 18910 half-hourly GPS positions in total. GPS success that is the percentage of successful positioning attempts per data set, averaged 81% (range 29–99%). We restricted our analysis of travel speed to half-hourly positions only and used positions at hourly intervals for all other analyses. In order to detect prey remains, we created 100 m buffers around all positions (Figure 2a). We dissolved the area of overlapping buffers and defined them as clusters (Sand et al. 2005, 2008; Zimmermann et al. 2007). Clusters included not only consecutive positions but also revisits to the same spot over the entire study period. In this way, we classified 10951 hourly positions as wolf cluster positions. The other 2237 hourly positions were single positions further than 200 m from the next nearest position (Figure 2a). This method of spatially clustering positions at a given buffer radius of 100 m has proven to be the most successful for detecting prey remains in Scandinavia (Sand et al. 2005) and has since been applied in all Scandinavian kill rate studies (Zimmermann et al. 2007; Sand et al. 2008, 2012).

All cluster positions and most of the single positions, that is >12000 positions in total, were visited in the field a few days after the wolf was present and searched for prey remains with the help of dogs (Sand et al. 2008). This resulted in the detection of 315 wolf kills, of which 250 (79%) were associated with clusters of at least two positions (kill clusters), 39 (12%) were at single positions, and 26 (8%) were further than 100 m from the closest GPS-position. Clusters containing a den or rendezvous site, that is sites where cubs were moved to and placed during the summer (Murie 1944; Jędrzejewski et al. 2001), were identified by a star-formation movement pattern of the adult wolves and were not visited in the field until at least 1 week after wolves had left the area. They consisted of a minimum of 44 hourly positions. We categorized all other clusters as bed clusters, associated with resting behavior. Bed clusters consisted of a maximum of 36 hourly positions.

### Resource maps

As the wolf population was cross-border, we joined digital maps of Norway and Sweden. Vector data of roads and houses were derived from the Norwegian (scale 1:50000) and Swedish (1:100000) national maps. We categorized roads as either gravel roads (“Enskilda vägar” in Sweden and “Privatvei” in Norway) or main roads (“Allmäna vägar” in Sweden and “Riksvei,” “Fylkesvei” and “Kommunal vei” in Norway). In general, main roads were paved. Due to the large size of the study area (Figure 1), we were not able to measure human activity on the >2400 km of main roads and >11300 km of gravel roads included in this study (Table 1). The classification of roads is based on the assumption that main roads connect human settlements, implying higher and more regular human disturbance. Gravel roads are mostly logging roads that experience short-term heavy use during logging or thinning operations once every 5–50 years, and moderate to low use during the fall game hunting season. The unified house map included all types of human buildings in both countries.

Land cover data were derived from satellite-based maps that had been classified and approved by national authorities in both countries. In Norway, the SatVeg raster data (Source: Norwegian Environment Agency) had a pixel size of 30 m. The Swedish Corine Land Cover map with a pixel size of 25 m was provided by the National Land Survey of Sweden. For this study, we simplified the original habitat classes (24 in Norway and 59 in Sweden) into four land cover categories: Forest, Water, Mire, and Open. The last class included built-up areas, agricultural fields, and other terrestrial, nonforested areas.

### Wolf travel speed

We calculated wolf travel speed from individual steps with a maximum time length of 35 min and minimum step length of 200 m, resulting in a total of 3188 steps from 25 data sets. We categorized start and end positions of each step as being either on or off of a road, within a threshold of 30 m. The distance of 30 m corresponds to the inaccuracy of GPS positions (Bowman et al. 2000; Rodgers 2001; Cargnelutti et al. 2007). In this way, we may have misclassified some wolf positions in close vicinity to roads as being on the road. Still, we considered this distance to be close enough for wolves to be aware of the road and to use it or the ditch as a guide through the landscape. For each step, we grouped the class variable Road into one of three states: 1) start AND end on a road; 2) either start OR end on a road; and 3) start AND end off road. In addition to this variable, we included the reproductive status of the wolves (Reproduction, Breeding, or Nonbreeding) and a two-class variable Time of day (Day 08:00–19:59; Night 20:00–07:59) in a linear mixed model (LMM, R 2.13.2). The classes of Time of day were more connected to human activity level than light conditions. During the summer months, nights do not get completely dark in the study area. Data set-ID (i.e. unique individual-study period combination) was nested within territory as a random factor and travel speed was the response variable. We eliminated nonsignificant (*P* > 0.05) variables and interactions in a stepwise backward procedure. For the final model, we reported the marginal and conditional *R* ^2^ for LMMs as proposed by Nakagawa and Schielzeth (2013).

### Wolf movement at the site scale: step selection functions (SSFs)

For the site level habitat selection study (fourth order, Johnson 1980), we applied a matched case-control design (Whittington 2002; Boyce et al. 2003; Whittington et al. 2005). By contrasting the resources at each animal position with a set of paired random points, we could estimate whether and to what extent certain resources affected animal movement. This design is statistically solved using conditional logistic regression (Whittington 2002; Boyce et al. 2003; Craiu et al. 2008). Fortin et al. (2005) coined the term SSF where the creation of random points or steps is based on step characteristics of the animal path, rather than on the animal positions per se, as proposed for Resource Selection Functions (RSFs) (Manly et al. 2002).

For the SSF analyses, we categorized steps between hourly positions with a minimum length of 200 m into traveling (from single to single hourly positions), travel-to-cluster, cluster-to-travel and cluster-to-cluster steps (Figure 2a). We excluded steps to and from den and rendezvous clusters to avoid spatial autocorrelation, resulting in a total of 3154 steps. Frequency distributions of step length and turning angle differed among step categories (Figure 2b), with traveling steps being longer and more linear, and cluster-to-cluster steps being short and having the highest diversity of turning angles. Due to these differences, we used the frequency distributions of turning angles and step lengths to create 10 random steps per real step for each step category separately, using the conditional point sampling tool of Hawth’s tools (Beyer 2004). A 10:1 ratio between paired random and real steps has been successfully applied in other earlier studies (Whittington 2002; Whittington et al. 2005; Coulon et al. 2008). The 10 random steps together with the real step are called a stratum in the statistical language. We calculated the distances to the closest road and house, and determined the habitat type for the end points of each real and random step of each stratum. If a random step ended in water, we assigned the closest terrestrial habitat type to this end point.

The response variable of the step selection models was a binary term with 1 for the used wolf locations, that is the end points of the real steps, and 0 for the end points of the random steps. To match the random steps to the corresponding real step, we applied conditional logistic regression (R 3.0.0) following the approach chosen by Fortin et al. (2009). Due to expected autocorrelation within territories, we used generalized estimating equations (GEE) including territory in the cluster term of the coxph-command (R package survival, Therneau 2014) to create robust standard errors. We preferred GEE to general linear mixed models (GLMM) because the sample size varied between individuals, and we were interested in the marginal rather than conditional estimates, that is drawing inferences for the entire Scandinavian wolf population rather than analyzing the differences between the studied individuals (Koper and Manseau 2009).

We estimated SSFs for all steps compiled, and separately for all combinations of time of day and behavioral state, that is at the end point of the real step the wolf was either handling prey (Kill), resting at a bed cluster (Rest), or traveling (single position, Travel) (Figure 2a). We ran an initial set of SSF models that included only the predictor on (<30 m) or off road (>30 m from closest road). Cross-validation of this set of SSFs was not possible because there was only one predictor and it had just two classes (on or off road). We however present the number of observed and random steps ending on roads.

With the second set of SSF-models, we tested whether distance to human infrastructure (roads, houses) and land cover type predicted the choice of where wolves ended a step. Here, we included a quadratic term for distance to infrastructure to test for selection of intermediate distances. After checking predictor variables for collinearity with a pairwise Pearson rank correlation, we started with a full model that included all uncorrelated (*r* < 0.60) predictors and subsequently eliminated nonsignificant (*P* > 0.05) predictors in a stepwise backward procedure. We cross-validated the final models by excluding one wolf territory at the time, estimating model coefficients for the retained territories, and using these coefficients to predict the SSF for all real and random steps of the excluded territories. The predicted SSFs were ranked within each stratum of 11 paired steps with ranks from 1 to 11. From each stratum, we randomly sampled one random step. We then used a paired Wilcoxon signed rank test to validate whether the ranks of the real steps were higher than the ranks of the random steps. If *P* > 0.05, model fit was regarded as insufficient and the model was rejected.

### Wolf habitat selection at the patch scale: RSFs

To test whether the wolves selected patches with low road densities within their home ranges (third order habitat selection, Johnson 1980), we applied RSF-models with a presence/available design (Manly et al. 2002). The home range was defined as the 100% minimum convex polygon (MCP) of all GPS positions in each of the 33 data sets. Used patches were considered to be all single positions and the first position in time of each cluster, in total 3291 positions, buffered with 1.78 km, resulting in a circular patch of 10 km^2^. As the mean number of used patches per data set was 99.7, we generated 100 random patches of equal dimensions (10 km^2^) within each home range to describe habitat availability.

For each used and random patch, we derived the density of gravel and main roads (km roads/km^2^), the density of houses (km^−2^), and the percentage availability of the different land cover types. These variables were the fixed factors in mixed effects logistic regression models (GLMM), and we included the data set-ID nested within territory as a random factor. The response was a binary term of 1 for used and 0 for available patches. We started with a full model including all noncorrelated (Pearson’s *r* < 0.6) predictor variables in linear and quadratic form and used stepwise backward selection to exclude nonsignificant variables (*P* > 0.05). We did this modeling procedure separately for breeding and nonbreeding wolves in combination with the three different behaviors (handling kills, resting, and traveling).

We used 10-fold cross-validation to validate our models (cf. Boyce et al. 2002; Houle et al. 2010). For each training set, we extracted the model coefficients of the fixed effects and used them to predict the RSF values of the corresponding validation set. The validation set was then sorted by the RSF and split into 10 equal-sized bins. For each bin, we calculated the relative frequency of used patches. The degree of correlation (Spearman rank correlation) between the rank of the bin and the relative frequency of used patches was used as an indicator of model fit. We repeated this process 100 times for each final model and rejected models with an average Spearman’s *r* < 0.6.

### Functional response of road use at the home range scale

We explored the relationship between road availability and use at the home range scale (second order, Johnson 1980) separately for breeding and nonbreeding wolves as home range use differed strongly with reproductive status. Nonbreeders ranged over areas 2.2 times larger than breeders (average ± *SE* home range size (100% MCP) of nonbreeders 818±138 km^2^; breeders 377±39 km^2^; *t* = 3.062, *P* = 0.009).

For gravel road availability, we created 30 m buffers along all gravel roads and calculated the proportion of the land area covered by the buffered roads within individual home ranges. Gravel road use was the proportion of hourly positions per data set within 30 m of the closest gravel road. We ran linear regression models of the proportion of wolf positions on gravel roads, with proportion of gravel road area as the main predictor. If wolves used gravel roads in proportion to their density, we would expect a linear relationship with slope = 1 and intercept = 0. In addition we added the following covariates into the full model: Home range size, median Julian date of the study period, and sex of wolves. We eliminated nonsignificant variables (*P* > 0.05) in a stepwise backward procedure.

## RESULTS

### Wolves traveled faster on than off roads

While traveling off road, wolves had an average speed of 2.15 km/h [*n* = 2500 steps, standard deviation (*SD*) = 1.54 km/h]. On roads, wolves traveled on average 1.8 times faster at a speed of 3.84 km/h (*n* = 91 steps, *SD* = 1.53 km/h). Speed was intermediate if wolves traveled partly on roads (*n* = 597 steps, mean = 2.96 km/h, *SD* = 1.41 km/h). The final LMM of travel speed included the variable Road in interaction with Reproduction, and Time of day (Table 2). Marginal and conditional *R* ^2^_LMM_ of this model were 7.6% and 15.5%, respectively. Breeding wolves moved on average 1.23 times faster than nonbreeders, with the difference being most pronounced when wolves used roads. Breeders had an average speed of 4.04 and 3.75 km/h on roads at night and day respectively, as compared to 3.15 and 2.86 km/h for nonbreeders. Speed was similar for male and female wolves, and sex was not retained in the final model.

**Table 2:** Model estimates of the final model for wolf travel speed (km/h) in Scandinavia, based on *n* = 3188 half-hourly steps

	Beta	*SE*	*t* value	*P* value
Intercept	2.863	0.295	9.720	<0.001
Road (off road)	−1.298	0.220	−5.888	<0.001
Road (partly)	−0.370	0.228	−1.622	0.105
Reproduction (breeding)	0.886	0.392	2.260	0.045
Time of day (night)	0.288	0.058	5.000	<0.001
Road (off road):reproduction (breeding)	−0.513	0.308	−1.663	0.096
Road (partly):reproduction (breeding)	−0.836	0.321	−2.603	0.009

### To what extent did wolves use roads?

Of all 3154 hourly steps used in the SSF models, 328 (10.4%) ended on gravel roads and 30 (1.0%) on main roads. Taking into account road availability in the SSF-models, wolves were 3.1 times more likely (e^β^, Table 3) to end a step on a gravel road than off road (*P* < 0.001), but there was no such preference or avoidance of main roads (*P* = 0.097). The separate SSFs for different times of day and behavior revealed that wolves preferred to use roads of either type for traveling, but not for other behaviors. They were 3.5 and 5.2 times more likely to travel on gravel roads during day and night, respectively (e^β^, Table 3), than off roads. Main roads were only preferred for travel during night time, with wolves being 1.9 times more likely to travel on a main road than off road (*P* = 0.002, Table 3).

**Table 3:** SSFs of Scandinavian wolves for the different combinations of time of day and behavior, together with number of real and random steps ending on a gravel road or a main road, and a summary of the conditional logistic regression models with robust *SE* estimation

Model	*n* steps	Gravel road					Main road				
	Total	# Real steps	# Random steps	β	*SE*	*P*	# Real steps	# Random steps	β	*SE*	*P*
All steps	3154	328	11499	1.137	0.127	<0.001	33	243	0.313	0.188	0.097
Day kill	142	4	60	−0.427	0.622	0.493	0	7	−15.150	0.553	<0.001
Day rest	323	7	120	−0.558	0.294	0.055	0	15	−15.150	0.427	<0.001
Day travel	481	57	186	1.243	0.215	<0.001	4	31	0.260	0.417	0.534
Night kill	361	14	130	0.078	0.278	0.780	0	23	−16.180	0.389	<0.001
Night rest	455	19	145	0.284	0.349	0.416	3	27	0.106	0.686	0.877
Night travel	1392	227	508	1.647	0.143	<0.001	26	140	0.639	0.208	0.002

The coefficients (β) are the logs of the odds ratio for selecting a road versus being off road. For each real step, there are 10 matched random steps. For comparison of number of real and random steps on roads, number of random steps needs to be divided by 10.

### Distance to human infrastructure

For each combination of time of day and behavior, the distance to the closest gravel road and/or main road was a significant predictor of the SSF (Table 4). However, model validation revealed a poor model fit for SSFs of wolves moving to kill sites (Table 4), and we are therefore unable to conclude whether and how wolves chose kill sites in relation to human infrastructure.

**Table 4:** Coefficients (β) and robust *SE* of SSFs for wolves in Scandinavia in different behavioral states (handling prey [kill], resting and traveling) during day and night hours

Time of day	Behavior	*N* steps	Gravel road		Main road		House		Habitat in rel. to Forest		Cross-validation	
			Distance	Distance^2	Distance	Distance^2	Distance	Distance^2	Mire	Open	Wilcox V	*P*
Day	Kill	142			0.358** ± 0.136	−0.034* ± 0.014					5288	0.280
	Rest	323	0.293±0.187	−0.193** ± 0.074	0.149*** ± 0.044		1.478*** ± 0.253	−0.403*** ± 0.119	−0.170±0.237	−0.555** ± 0.215	34528	<0.001
	Travel	481			0.191*** ± 0.038		0.707** ± 0.219	−0.277** ± 0.094	−0.010±0.128	−1.099*** ± 0.321	72454	<0.001
Night	Kill	361			0.335*** ± 0.076	−0.034** ± 0.013					33974	0.226
	Rest	455			0.102* ± 0.040				−0.299±0.198	−0.812* ± 0.408	56369	0.037
	Travel	1392	−1.008*** ± 0.196	0.306*** ± 0.086	0.159*** ± 0.039	−0.017*** ± 0.004					553174	<0.001

Cross-validation confirmed four and rejected two SSFs at the alpha-level of 0.05.

**Figure 3:**
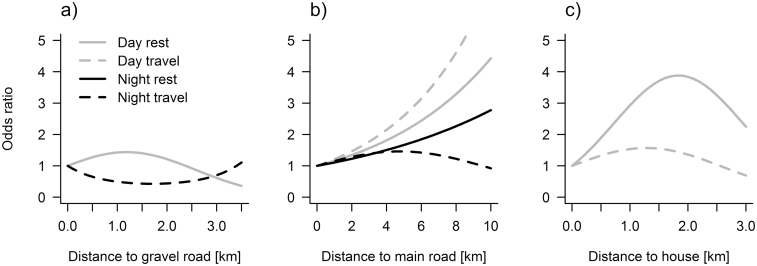
SSFs for Scandinavian wolves during summer, expressed as odds ratios (e^β^) in relation to distance to gravel roads (a), main roads (b) and houses (c). Estimates of βs are given in Table 4.

While resting during day time, wolves preferred intermediate distances to gravel roads, and they were 1.4 times more likely to bed at distances of 1–1.5 km from the closest gravel road as compared to directly at the road (Figure 3a). They preferred to have day bed sites far away from main roads (Figure 3b) and at intermediate distances to houses, with a peak at 2 km from the closest house (Figure 3c). In addition, they avoided open habitats for day bed sites (Table 4). While resting during night time, they also avoided open habitats and preferred areas far from main roads, although this preference pattern was weaker than during the day time (lower odds ratios in Figure 3a and significance of coefficients in Table 4). Gravel roads did not seem to affect the choice of resting sites during night time.

While traveling during day time, wolves preferred areas far from main roads (Figure 3b) and at intermediate distances to houses (Figure 3c), whereas they seemed to be indifferent to gravel roads. They avoided open habitats during day time travel but were indifferent to the habitat type during night time travel (Table 4). The strong preference of wolves for using gravel roads for traveling during night time (Table 3) is reflected by the U-shaped odds ratio in Figure 3a, indicating preference for near and far distances to gravel roads. While traveling during night time, wolves showed a weak preference for intermediate distances to main roads (Figure 3b, Table 4).

### Patch selection as a function of house density

**Figure 4:**
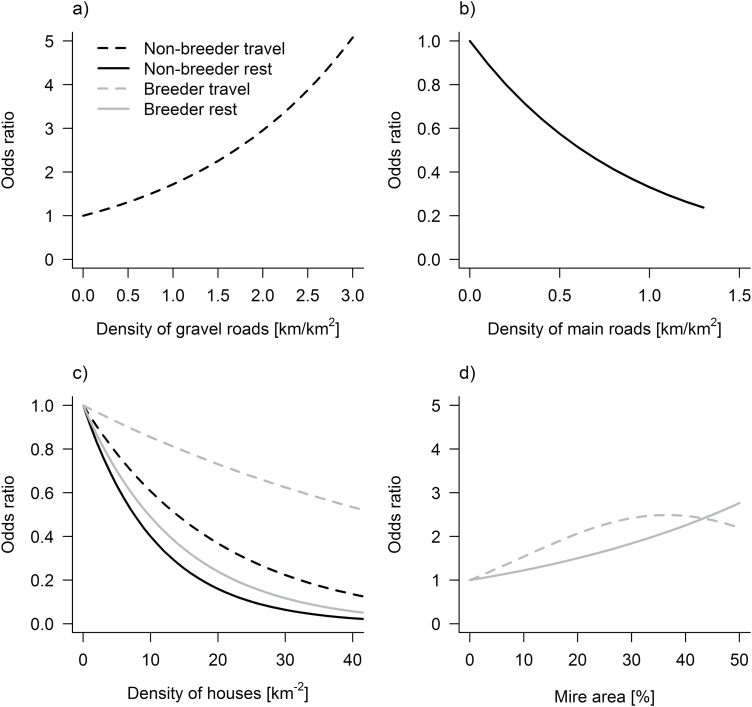
Resource selection functions for patch (10 km^2^) selection of Scandinavian wolves during summer, expressed as odds ratios (e^β^) in relation to density of gravel roads (a), main roads (b), houses (c), and the proportion of the patch covered with mires (d). Estimates of βs are given in Table 5.

The RSF-models developed to predict patch selection for kill sites of breeding and nonbreeding wolves did not have sufficiently good fit to draw conclusions about the potential effects of roads and houses on kill site selection (Table 5). Nonbreeding wolves were most likely to travel in patches with high gravel road densities (Table 5), and a patch was 1.7 times more likely to be used (e^β^) if the gravel road density was increased by 1 km/km^2^ (Figure 4a). Nonbreeders selected patches with low main road densities to rest in (Table 5), and the likelihood of using a patch for resting was reduced by one-third if the main road density increased by 1 km/km^2^ (Figure 4b). In contrast, road densities did not relate to patch selection of breeding wolves. House densities were negatively correlated with the likelihood of wolves using a patch for resting or traveling, regardless of their reproductive status (Table 5, Figure 4c). This negative relationship was stronger if wolves were resting than if they were traveling. The likelihood of breeding wolves using a patch for resting or traveling increased with the proportion of the patch covered by mire (Table 5, Figure 4d).

**Table 5:** Coefficients (β) ± *SE* of RSF models describing the patch selection (patch size = 10 km^2^) of Scandinavian wolves within their home ranges during summer

Reproductive status	Behavior	*N* positions	Gravel road km/km^2^		Main road km/km^2^	Houses km^−2^	Mires		Cross-validation
			Density	Density ^2	Density	Density	Proportion	Proportion ^2	*r*
Breeders	Kill	273	0.953±0.545	−0.597* ± 0.298					−0.026±0.017
	Rest	392				−0.071*** ± 0.018	2.033*** ± 0.578		0.745±0.007
	Travel	1492				−0.016* ± 0.008	4.996*** ± 1.076	−6.838** ± 2.524	0.793±0.006
Nonbreeders	Kill	102				−0.136** ± 0.049			0.466±0.013
	Rest	313			−1.107** ± 0.396	−0.092** ± 0.030			0.739±0.008
	Travel	719	0.542*** ± 0.140			−0.050** ± 0.015			0.704±0.007

Cross-validation results indicate sufficient model fit (Spearman’s *r* > 0.6) for four of the six combinations of reproductive status and behavior.

### Functional response to gravel road density

**Figure 5:**
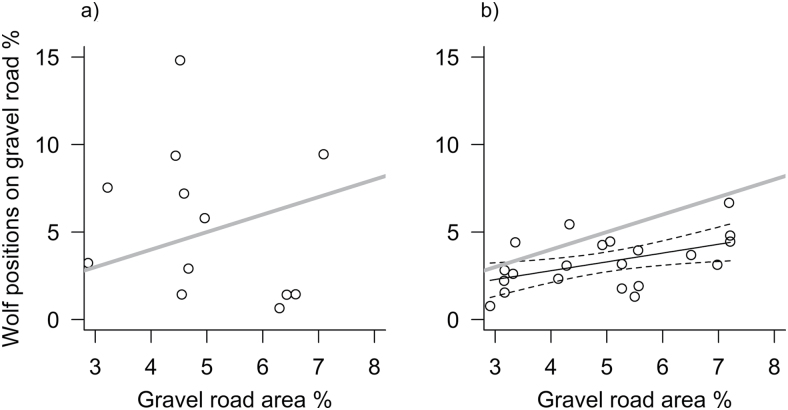
Gravel road use, expressed as % wolf positions on gravel roads, in relation to gravel road availability in the home ranges of Scandinavian wolves, expressed as % of land area covered with gravel roads, for nonbreeding (a) and breeding (b) adult wolves. The gray line indicates road use in proportion to availability (slope = 1). Black lines in (b) indicate the slope and 95% confidence limits of the linear model of road use and availability.

Gravel roads with a 30 m buffer covered on average 5±0.5% (2 *SE*, range 2.9–7.2%) of the land area of the home ranges (*n* = 33). Road use varied highly among nonbreeding wolves (*n* = 12), with on average 4.4±2.5% (range 0.7–14.8%) of hourly positions on gravel roads (Figure 5a). There was no correlation between road availability and road use by nonbreeding adult wolves (*P* = 0.499, Figure 5a). However, road use by breeding wolves (*n* = 21) was less variable (mean = 3.3±0.6%) and positively related to road availability (Figure 5b). The proportion of gravel road area was the only significant variable in the final linear model describing this relationship (Intercept = 0.779±1.035; slope = 0.504±0.201; *P* = 0.021; *R* ^*2*^ = 0.25). The slope was significantly lower than 1 (Figure 5b) indicating that although breeding wolves increased road use with increasing gravel road availability in their home range, this increase was 50% lower than expected. Sex of wolves, home range size, and Julian date were not related to road use by breeding or nonbreeding wolves.

## DISCUSSION

We have demonstrated that the behavioral response of Scan dinavian wolves to roads is a complex multi-factorial process dependent on time of day, road type, behavioral state, reproductive status, and spatial scale. In the discussion below, we break this complexity down by considering each spatial scale from site level to patch level and finally to the home range level. Special emphasis is given to the differences between breeding and nonbreeding wolves, as reproductive status is an important determinant of population viability and has not previously been studied in comparable wolf-road publications.

At the site scale, wolves in Scandinavia showed a clear preference for traveling on gravel roads and even on main roads during night hours, in summer. Similar preference patterns of wolves for minor roads and other man-made linear features have been described elsewhere (Whittington et al. 2005, 2011; Houle et al. 2010; Gurarie et al. 2011). Ease of travel is the most plausible reason why wolves in Scandinavia displayed this strong preference. They traveled nearly twice as fast on roads compared to off roads and breeding wolves traveled faster than nonbreeders, especially on roads. When food has to be provided to other pack members at den or rendezvous sites, roads likely serve as a positive medium for traveling in terms of minimizing energy expenditure and maximizing speed of food delivery. Higher travel speeds on linear features have also been recorded for wolves collared with Very High Frequency (VHF) tags in Poland (Musiani et al. 1998) and GPS-collared wolves on seismic lines in Alberta, Canada (James 1999) while GPS-collared cougars (*Puma concolor* L.) (Dickson et al. 2005) and bison (*Bison bison* L.) (Bruggeman et al. 2007) traveled faster on dirt roads than off road.

Territory maintenance by scent-marking, which allows efficient communication toward intruders, is another plausible explanation for the extensive use of roads by wolves. Wolves regularly scent-mark along roads (Zub et al. 2003; Barja et al. 2004), and have higher scent-marking frequencies on roads than off roads (Peters and Mech 1975). An alternative explanation is that prey use roads for travel and roadside or post-logging vegetation for food and minerals (Laurian et al. 2008; Hebblewhite et al. 2009), which in turn attracts the wolves. However, an earlier case study in Scandinavia showed that moose with calves, the main prey of wolves in this area, avoided being close to gravel roads during summer (Eriksen et al. 2009), perhaps as an anti-predator strategy against wolves.

Although at the site scale Scandinavian wolves displayed an overall preference for roads during summer, this preference was dependent on the road type, with main roads being less attractive than gravel roads. These findings support results from other studies that showed that wolves decreased use of roads and paths or increased their distance from these features with increasing size of roads or increasing rate of human use (Kunkel and Pletscher 2000; Kaartinen et al. 2005; Whittington et al. 2005; Theuerkauf et al. 2007; Gurarie et al. 2011; Muhly et al. 2011; Rogala et al. 2011). Time of day influenced the behavioral response of wolves to human infrastructure, likely caused by the diurnal activity pattern of humans. A similar night-bias of road use has been reported for wolves in Europe and Canada (Blanco et al. 2005; Theuerkauf et al. 2007; Hebblewhite and Merrill 2008) and a meta-analysis across wolf studies concluded that nocturnal activity and movement were positively correlated with public road density (Theuerkauf 2009).

Resource selection is a function of the behavioral state of an animal (Beyer et al. 2010). Although the wolves in our study preferred using roads for traveling, they preferred to rest at intermediate distances to gravel roads and far away from main roads. We interpret the observed preference of resting at intermediate distances to gravel roads as a trade-off between the risk of encountering humans and good access to roads for increased travel speed and scent-marking.

Minimizing the probability of encountering humans has been identified as an important factor shaping habitat selection of wolves within the home range, and road density has been used as a proxy for this (Ciucci et al. 1997; Whittington et al. 2005; Gurarie et al. 2011). However, our prediction that road densities at the patch level would affect wolf habitat selection negatively was only partly supported. Nonbreeding wolves preferred to rest in patches with low main road densities while contrary to our prediction they were more likely to travel in patches with high gravel road densities. For breeding wolves, we could not detect any relationship between patch selection and road density. More importantly however, patch selection by wolves was negatively related to house densities within the home range, both for breeders and nonbreeders, when resting and traveling. Theuerkauf et al. (2003a) described a similar relationship between the number of inhabitants of settlements and avoidance of close surroundings by wolves in Poland. At this regional scale, houses may be a more predictable indicator than roads of the probability of encountering humans. Human activity along gravel roads depends on the land use cover and history in the area (Thurber et al. 1994; Houle et al. 2010), whether the road has restricted access for motorized traffic, and whether it connects with major traffic arteries and settlements. These are factors that we did not measure or account for in our study. Another indicator of the probability of encountering humans is the ratio between productive and unproductive lands in a patch. As the proportion of the patch area covered by mire (unproductive land) increases, we expect a decrease in human activity. In our study, preferred patches of the home range of breeding wolves had a relatively high proportion of mire.

Finally, we expected gravel road use by wolves to vary with gravel road density across home ranges. This functional response of wolves to roads was only supported for breeding wolves in Scandinavia. Although road use by breeders was positively correlated with gravel road density, the functional response was less than proportional to gravel road availability, that is breeding wolves used gravel roads less frequently than expected as gravel road densities increased. In a study of two wolf packs tracked on snow in Alberta, Canada, Whittington et al. (2005) found that the use of roads and trails was negatively coupled with road density. Their study looked at the functional response at the patch scale within the home range. Another study of GPS-collared wolves in Quebec, Canada, found selection of forest roads decreased with increasing forest road density within the home range, while at the between-pack scale, road density was not related to road use (Houle et al. 2010). RSF-models of GPS-data from a wolf study in Alberta revealed a more complex picture of the selection of proximity to human activity (Hebblewhite and Merrill 2008). Although wolf packs in areas of low human activity were indifferent to proximity to humans, wolves from a few packs in areas of high human activity showed a pattern of selection for close proximity to man-made linear features that varied with season and time of day. Hebblewhite and Merrill (2008) attributed this functional response at the pack scale to the constraints of sharing habitats with humans. Wolf avoidance of areas with high road or house densities, either directly at the patch scale or relatively at the home range scale may result in a trophic cascade. Lowered predation pressure aggregates prey, leading to increased browsing pressure in areas of high human activity (Hebblewhite et al. 2005b; Beyer et al. 2007).

Wolves in Scandinavia have adapted to use roads for traveling, scent-marking, and territorial patrolling, but they have also developed cryptic behavioral responses to roads, likely driven by the increased risks associated with human presence. The high behavioral plasticity which allows such ambivalent responses of wolves toward infrastructure is a key factor in the recent wolf recovery in industrialized countries, many of which have higher densities of roads and humans than in Scandinavia. There are other success stories of species that have adaptively responded to man-made habitat alterations while still avoiding increased rates of human-caused mortality, for example urban wildlife or raptors feeding on vehicle-killed prey (Tuomainen and Candolin 2011; Francis and Chadwick 2012). However, there are many more examples of species that have maladaptive responses, causing decreased individual fitness with negative effects on population growth and distribution (Fahrig and Rytwinski 2009; Tuomainen and Candolin 2011). Migration and connectivity in Scandinavian wild reindeer have been interrupted by the barrier effect of linear features (Vistnes et al. 2004). In North America, the closure or removal of logging roads and other measures to decrease human access have been proposed to restore grizzly and black bear (*Ursus arctos* L.*, Ursus americanus* Pallas) habitat by limiting the mortality risk of hunting and poaching (Nielsen et al. 2006; Switalski and Nelson 2011). Despite the seemingly well-functioning adaptation of wolves to man-made habitat alterations, we should be aware that roads may interact with human attitudes, enabling increased human-caused mortality of wolves. The acceptance of wolf poaching is relatively high in rural Scandinavia (Gangaas et al. 2013), and the accessibility of wolf territories by gravel roads is crucial for poachers to increase their encounter rate with wolves, especially during the breeding period. The existing network of gravel roads is likely to be an important factor governing the vulnerability of wolves to human caused mortality and may negatively affect the resilience of the relatively small Scandinavian wolf population which currently suffers from inbreeding depression (Liberg et al. 2005; Bensch et al. 2006).

## FUNDING

This SKANDULV study was supported by the Norwegian Research Council, the Norwegian Directorate of Nature Management, the Norwegian Institute for Nature Research, the County Governor of Hedmark, the Swedish Environmental Protection Agency, World Wildlife Fund for Nature (Sweden), Swedish University of Agricultural Sciences, the Swedish Association for Hunting and Wildlife Management, the Swedish Carnivore Association, several county municipalities, and Hedmark University College.
